# The Year Book of Treatment for 1898

**Published:** 1898-05

**Authors:** 


THE YEAR BOOK OF TREATMENT FOR 1898.—A critical review for practitioners of medicine and surgery. Contributors: Francis D. Boyd, M. D., Patrick Manson, M. D., Dudley W. Buxton, M. D., Malcolm Morris, F. R. C. S., Albert Carless, M. S., London; Edmund Owen, F. R. C. S., Alfred Cooper, F. R. C. S., Sydney Phillips, M. D., Geo. P. Field, M. R. C. S., Henry Power, F. R. C. S., Archibald E. Garrod, M. D., E. S. Reynolds, M. D., G. A. Gibson, M. D., William Rose, F. R. C. S., M. Handfield Jones, M. D., Gustav Schorstein, M. B., Riginald Harrison, F. R. C. S., St. Clair Thompson, M. D., Herbert P. Hawkins, M. D., Nestor Tirard, M. D., G. Ernest Herman, M. D., W. J. Walsham, F. R. C. S., J. Ernest Lane, F. R. C. S., E. F. Willoughby, M. D., A. P. Luff, M. D., Dawson Williams, M. D. Published by Lea Bros. & Co., Philadelphia and New York. Cloth, 488 pages; price, $1.50.

The publishers' announcement of this work states that it furnishes a critical and trustworthy epitome of a year's progress in all branches of medicine and surgery. That it has done so acceptably is demonstrated by the fact that fourteen consecutive annual issues have been demanded. The work is systematically arranged and thoroughly indexed, making its contents conveniently accessible to the reader.

Much attention in detail is given to the new therapeutics by serum, and we are impressed by the perusal of this and similar recent publications with the idea that while nothing very practical may have yet been discovered along these lines, that the tremendous activity of our investigators will not be altogether wasted, and that much useful information will arise from their labors. Koch has again returned to his investigations of tuberculin, and has evidently made progress, and we can but hope that this truly great man will yet emerge from the clouds of obloquy, bringing the shining jewels of truth with him.

For its compass, the work cannot be much improved upon.

